# Exploring Barriers and Facilitators for Cervical Cancer Screening in Iquitos, Peru: Application of the COM-B Behavior Model to Inform Program Implementation

**DOI:** 10.1200/GO.22.25000

**Published:** 2022-05-05

**Authors:** Margarita Correa-Mendez, Valerie Paz-Soldan, Batel Blechter, Patti E. Gravitt, Anne F. Rositch, Graciela Meza

**Affiliations:** ^1^ NCI, Rockville, MD; ^2^ Tulane University School of Public Health and Tropical Medicine, New Orleans, LA; ^3^ Johns Hopkins Bloomberg School of Public Health, Baltimore, MD; ^4^ Facultad de Medicina Humana, Universidad Nacional de la Amazonía Peruana, Iquitos, Peru

## Abstract

Cervical cancer is the second cause of death among Peruvian women. In Iquitos, Proyecto Precancer engages key stakeholders to develop interventions that address women's needs and prevent cervical cancer. Successful prevention programs rely on behavior change interventions that must be supported by a theoretical understanding of the behavior. The Capability, Opportunity, Motivation, Behavior (COM-B) model informs what must be modified for a behavior change intervention to be effective.

**METHODS:**

A cervical cancer prevention focused Knowledge-Attitudes-Practices (KAP) survey was administered in 2017 to women ages 18-65 years living in Iquitos, Peru using census-based random sampling. To identify context-specific barriers and facilitators for cervical cancer screening in Iquitos, we mapped KAP survey data onto key themes in the COM-B model.

**RESULTS:**

Out of 619 women, 67.9% had been screened for cervical cancer at least once. Facilitators to screening-seeking behaviors were having the knowledge about cervical cancer (80.5% of women), partner support (74.8%), attending a health post (79.5%) and receiving information from a health post (63.6%). Among women not previously screened, fear was the primary barrier (41.7%). Perceived risk of cervical cancer was high (79.1%) but experiencing symptoms as a reason to seek screening remains a common misconception (36%).

**CONCLUSION:**

Behavior change interventions must address the sources of fear and raise awareness about seeking cervical cancer screening prior to symptom development. Focusing on health posts and partners as key facilitators could help improve the outcomes of programs implemented by Proyecto Precancer in Iquitos.

